# Sensitivity to Heavy-Metal Ions of Unfolded Fullerene Quantum Dots

**DOI:** 10.3390/s17112614

**Published:** 2017-11-14

**Authors:** Erica Ciotta, Stefano Paoloni, Maria Richetta, Paolo Prosposito, Pietro Tagliatesta, Chiara Lorecchio, Iole Venditti, Ilaria Fratoddi, Stefano Casciardi, Roberto Pizzoferrato

**Affiliations:** 1 Department of Industrial Engineering, University of Rome Tor Vergata, 00133 Rome, Italy; Erica.Ciotta@uniroma2.it (E.C.); stefano.paoloni@uniroma2.it (S.P.); richetta@uniroma2.it (M.R.); 2 Department of Industrial Engineering INSTM and CiMER, University of Rome Tor Vergata, 00133 Rome, Italy; paolo.prosposito@uniroma2.it; 3 Department of Chemical Sciences and Technology, University of Rome Tor Vergata, 00133 Rome, Italy; pietro.tagliatesta@uniroma2.it (P.T.); ChiaraLorec@hotmail.it (C.L.); 4 Department of Chemistry, University of Rome Sapienza, 00187 Rome, Italy; iole.venditti@uniroma1.it (I.V.); ilaria.fratoddi@uniroma1.it (I.F.); 5 National Institute for Insurance against Accidents at Work (INAIL), Department of Occupational and Environmental Medicine, Epidemiology and Hygiene, 00078 Monte Porzio Catone, 00133 Rome, Italy; s.casciardi@inail.it

**Keywords:** carbon materials, heavy metals, sensors, spectroscopy, photoluminescence, quenching

## Abstract

A novel type of graphene-like quantum dots, synthesized by oxidation and cage-opening of C_60_ buckminsterfullerene, has been studied as a fluorescent and absorptive probe for heavy-metal ions. The lattice structure of such unfolded fullerene quantum dots (UFQDs) is distinct from that of graphene since it includes both carbon hexagons and pentagons. The basic optical properties, however, are similar to those of regular graphene oxide quantum dots. On the other hand, UFQDs behave quite differently in the presence of heavy-metal ions, in that multiple sensitivity to Cu^2+^, Pb^2+^ and As(III) was observed through comparable quenching of the fluorescent emission and different variations of the transmittance spectrum. By dynamic light scattering measurements and transmission electron microscope (TEM) images we confirmed, for the first time in metal sensing, that this response is due to multiple complexation and subsequent aggregation of UFQDs. Nonetheless, the explanation of the distinct behaviour of transmittance in the presence of As(III) and the formation of precipitate with Pb^2+^ require further studies. These differences, however, also make it possible to discriminate between the three metal ions in view of the implementation of a selective multiple sensor.

## 1. Introduction 

In the past decade, research on nanomaterials has undergone a huge development in many different fields such as medicine, biology, energy and sensors [1,2,3,4,5,6]. In particular, innovative carbon-based nanomaterials such as graphene quantum dots (GQDs) and graphene oxide quantum dots (GOQDs) have attracted much interest as stable, nontoxic, photoluminescent nanomaterials with possible applications in chemical and biological sensors, drug delivery, bio-imaging and energy conversion [7,8,9,10,11,12,13]. The physical origin of the photoluminescence (PL) from these carbon-based nanoparticles is still quite controversial and different mechanisms have been suggested, possibly acting together, mainly based on quantum confinement in sp^2^ domains with a strong influence from edge and oxygen-containing functional groups [14,15,16,17,18]. Nonetheless, several groups have reported a characteristic fluorescence with uniform and stable features in aqueous solution of GOQDs prepared by a variety of methods, either based on bottom-up or on top-down synthesis from graphite [19,20,21,22,23,24,25,26,27]. The spectral profile and peak-emission wavelength of this emission can relatively be controlled by tailoring dimensions, chemical functionalization and excitation wavelength. Most importantly, some recent studies [28,29,30,31,32,33,34,35] have demonstrated that the PL intensity is significantly quenched in the presence of certain heavy-metal ions (Hg^2+^, Fe^2+^, Cu^2+^, Pb^2+^), thus triggering the interest of researchers in view of applications to optical sensors for these elements. Heavy metals, in fact, can be harmful to the environment and very toxic for human health at concentrations exceeding specific, while controversial, threshold values [36,37]. For instance, the current World Health Organization limit for arsenic and for lead in drinking water is 10 μg/L, whereas for copper it increases up to 2 mg/L [38]. The traditional laboratory methods for heavy-metal detection—such as high performance liquid chromatography (HPLC) [39] inductively coupled plasma mass spectrometry (ICP-MS), flame atomic absorption spectroscopy (FAAS), atomic fluorescence spectroscopy (AFS) and graphite furnace atomic absorption spectroscopy (GFAAS) [28] are reliable and sensitive, but generally require expensive, complex and heavy instrumentation with highly skilled staff and time consuming procedures. Therefore, in search of portable, fast, user-friendly and cheap sensors, other methods have been investigated, based on nanostructured materials with assistance of lab-on-chip (LOC) technology, which include electrochemical and optical processes [40]. In particular, electrochemical sensors usually have advantages such as high sensitivity, low cost and ease of miniaturization. Nevertheless, they lack enough selectivity due to the limited anodic range and have short life-time [28,41]. Compared to the above techniques, optical methods have received great attention since they can offer high selectivity, stability, intrinsic operational simplicity and immunity against electrical disturbance. Moreover, optical response can be based on different sensing mechanisms such as fluorescence quenching, photoinduced electron transfer (PET), Forster energy transfer (FRET), colorimetric and ratiometric techniques [9,13,16,42,43] 

However, few experimental studies have been carried out so far on the optical response of GOQDs, and quite scattered and partially contradictory results have been reported, which seem to depend on the synthesis method and/or the experimental conditions. Moreover, little theoretical analysis has been carried out to explain the mechanism for PL quenching, especially if one considers the response of bare GOQDs, i.e., without any specific functional group added to the carbon structure with the purpose of binding a specific metal. For instance, Wang et al., who firstly demonstrated that GOQDs can be used to efficiently detect heavy metals, reported the selective response of GO nanosheets to the presence of Fe^3+^ ions in aqueous solution [29]. The nanosheets were synthesized with a typical top-down strategy by exfoliating graphite flakes trough a modified Hummers method and subsequent cutting to nanosheets with periodic acid. A much lower sensitivity was found to other heavy metals, such as Pb^2+^ and Cu^2+^, and the selective quenching to iron was explained with the special coordinate interaction between Fe^3+^ and phenolic hydroxyl groups located at the nanosheets edge. On the other hand, in GOQDs prepared with a similar modified Hummers method, Hao’s group [30] demonstrated efficient and selective detection of Cu^2+^ and no response to other 15 heavy metals (including Fe^3+^). Based on the Stern-Volmer plot, the authors suggested a combination of static and dynamic quenching (collisional deactivation). Chakraborti et al. [31], by using graphene quantum dots synthesized with a bottom-up method from citric acid, reported selective PL quenching in the presence of Hg^2+^ at pH 7. No response to other metals was observed except a very little interference of Fe^3+^. The PL quenching was found to be reversible and fluorescence could be recovered by addition of ethylenediaminetetraacetic acid (EDTA), due to the formation of the strong complexes that EDTA makes with the mercury ions. In another study with similar synthesis [32], a very efficient quenching effect to Hg^2+^ was observed but no sensitivity to Fe^3+^ was detected at all, while recovery of fluorescence was achieved by using cysteine. A more complex situation was found in two studies, both dealing with GQDs prepared by chemical oxidation of carbon fibers [33,35]. Gao’s group [35] observed multiple sensitivity to Co^2+^, Mn^2+^, Ni^2+^ and Cu^2+^. Only the PL quenching due to copper(II), however, could be efficiently recovered with the addition of cysteine, a more specific metal chelator. Liao et al. [33] found a quenching effect at pH 8.5 in the presence of Mn^2+^, Ni^2+^, Co^2+^ and Cu^2+^. This response could be reversed by addition of EDTA. Conversely, non-recovering quenching was found with Cr^3+^, Fe^3+^ and Ru^3+^. Electron transfer to partly filled *d* orbitals or complexation with layer aggregation were taken into account to explain the two cases, respectively. Finally, a selective response to Cr^6+^ was found at pH 4.5 in commercially available GQDs [34]. It should be pointed out that such a surprising and somehow puzzling variety of response, in some cases with contradictory results, was obtained in carbon quantum dots which should basically share a similar structure modelled on graphene, i.e., a carbon hexagonal lattice, possibly with different extension of sp^2^ domains and content of defects. This suggests that precisely the different defects and functional groups, such as –OH, CH_2_ and –COOH, could determine the different response to the metal ions.

Within this framework, we have recently investigated [44] a novel type of graphene-like quantum dots synthesized by low-temperature oxidation and unfolding of C_60_ buckminsterfullerene [45]. Due to the peculiar structure of buckminsterfullerene ÷ the carbon nanosheets synthesized with this technique have a lattice made of both hexagons and pentagons, which could give rise to a different configuration for the sp^2^ domains, the content and location of defects and the functional groups. These differences, in turn, could produce a new scenario for the response of the quantum dots to the surrounding environment. In this study, therefore, we have carried out a systematic characterization of the effects produced by some commonly encountered heavy-metal ions, Hg^2+^, Fe^2+^, Cu^2+^, Ni^2+^, Pb^2+^, Co^2+^, As(III), on the optical properties of such unfolded fullerene quantum dots (UFQDs). Monovalent Na^+^ was also investigated as control species. The experimental results have been compared to those reported for regular GOQDs. Fluorescence quenching and transmittance measurements have been correlated to dynamic light scattering (DLS) characterization in order to get a better understanding of the interaction mechanisms between carbon-based quantum dots and analytes. In addition, we have studied how the different optical response exhibited by UFQDs to three heavy-metal ions could make it possible to discriminate between the three species for the implementation of a selective optical sensor. 

## 2. Materials and Methods

### 2.1. Materials and Apparatus

Buckminsterfullerene (C_60_, TERM USA, Fort Bragg, CA, USA), sulfuric acid (H_2_SO_4_, 99.9%, Sigma Aldrich, Milano, Italy), sodium nitrate (NaNO_3_, Sigma Aldrich), sodium hydroxide (NaOH, Carlo Erba, Milano, Italy), potassium permanganate (KMnO_4_, Sigma Aldrich), and hydrogen peroxide (H_2_O_2_, Sigma Aldrich) were used as purchased without further purification. Metal salts used for studying the system sensitivity, purchased from Sigma Aldrich, are NiCl_2_ 6H_2_O, NaCl, CoCl_2_ 6H_2_O, CuCl_2_ 6H_2_O, Pb(NO_3_)_2_ 5H_2_O, Hg(NO_3_)_2_ H_2_O, NaAsO_2_, FeSO_4_ 7H_2_O). The metal salts solutions were prepared at the concentration of 1000 μM. For this work, deionized water obtained from a Milli-Q water purification system (Millipore, Burlington, MA, USA) was used. 

### 2.2. Preparation of UFQDs

UFQDs were prepared from C_60_ fullerene by a chemical oxidation through a modified Hummers method, as reported elsewhere [45]. Briefly, the fullerene was oxidized using NaNO_3_, KMnO_4_ and H_2_SO_4_. The solution was first placed in an ice bath and then stirred for 4 h at room temperature. During the final phase of reaction, variations of temperature and additions of water were made. The reaction was quenched with H_2_O_2_ and the pH was brought to 8 with NaOH 1 M. To remove all the residual compounds, the final UFQDs solution was obtained by dialyzing the solution in a dialysis bag (2 KDa) in deionized water for two days. After dialysis, the solution was further diluted 1:4 with deionized water to obtain what is therein referred to as the “stock UFQDs solution” at pH 7. The synthesis was performed six times on different days over a four-month time-period in order to test the reproducibility of the morphological and optical characteristics.

### 2.3. Instrumentation and Methods for the Response of UFQDs to Heavy-Metal Ions

FT IR spectra were routinely recorded on powders on a Perkin Elmer SpectrumOne spectrometer (Waltham, MA, USA). TEM measurements were performed in a FEI TECNAI 12 G2 (120 KeV) apparatus equipped with an energy filter (GATAN GIF model) and a Peltier cooled SSC (slow scan charged coupled device) multiscan camera (794 IF model). A droplet of the solution was used to disperse the UFQDs on a copper TEM grid (mesh 400) coated with ultrathin C carbon on holey carbon support. ^13^C-NMR spectra were recorded with a Bruker Avance 300 spectrometer (Bruker Corp., Billerica, MA, USA). The pH values of the solutions were checked with a digital pH meter (Hanna Instruments) calibrated with standard buffer solutions (Sigma Aldrich, Milano, Italy). UV-vis absorption spectra were recorded with a Cary 50 spectrophotometer (Varian, Inc., Palo Alto, CA, USA) by using fused silica cuvettes with either 10-mm or 1-mm optical path. Photoluminescence (PL) emission spectra were recorded on a specific laboratory set-up equipped with a 200-W continuous Hg(Xe) discharge lamp (Oriel instruments, Stratford, CT, USA), an excitation 25-cm monochromator (Photon Technology International, Inc., BirminghamNJ, USA) and an emission 25-cm monochromator (Cornerstone 260, Stratford, CT, USA) equipped with specific excitation-rejection filters and a R3896 photomultiplier (Hamamatsu Photonics Corp., Bridgewater, NJ, USA) by using conventional 90° geometry on fused-silica cuvettes with an optical length of 10 mm [46]. The typical excitation wavelength of λ_ex_ = 300 nm was used. The emission curves were fully corrected for the spectral response of the apparatus that was calibrated over the spectral range from 300 nm to 800 nm with a reference black-body lamp and standard-fluorophore solutions. A spectral band-pass of 2.5 nm was used for both the excitation and emission monochromators. 

Dynamic light scattering (DLS) measurements were carried out on the UFQDs aqueous suspensions using a Zetasizer instrument (Malvern) at a temperature of 25.0 ± 0.2 °C. Correlation data have been acquired and fitted in analogy to our previous works [47,48,49]. All measurements were taken at least three times and the average value ± standard deviation was reported. DLS measurements of UFQDs alone and with metal salts were carried out as follows. The reference UFQD solution was prepared by adding 1.5 mL of the stock solution to the same volume of deionized water and the mixture was stirred for 60 s before the measurement. 

In order to characterize the response to metal ions, the reference (blank) UFQD solution was prepared by adding 1.5 mL of the stock solution to the same volume of deionized water. The mixture was stirred for 60 s and then the transmittance and PL spectra were recorded. 

For the measurements in the presence of metal ions: 1.5 mL of the stock solution were mixed with 1.5 mL of the metal-salt water solution at the appropriate concentration. The mixture was stirred for 60 s and then the transmittance and PL spectra were recorded. The pH of the solution was measured immediately before and after each measurement.

## 3. Results and Discussion

### 3.1. Optical Characterization of UFQDs

**Figure 1:**
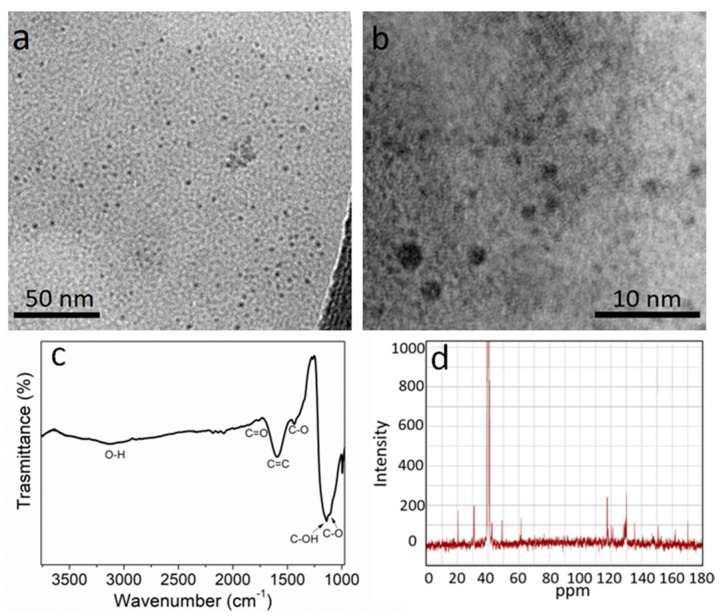
(**a**) Low-magnification and (**b**) high-magnification TEM image UFQDs. (**c**) FT-IR and (**d**) ^13^C NMR spectra of UFQDs.

**Figure 2:**
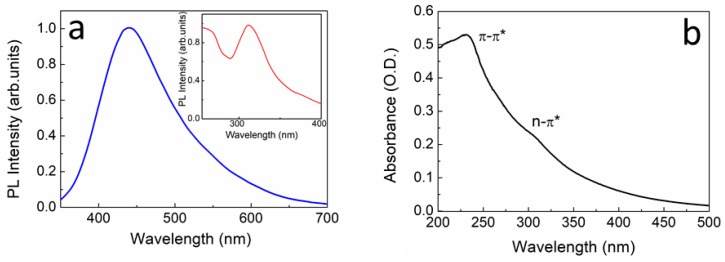

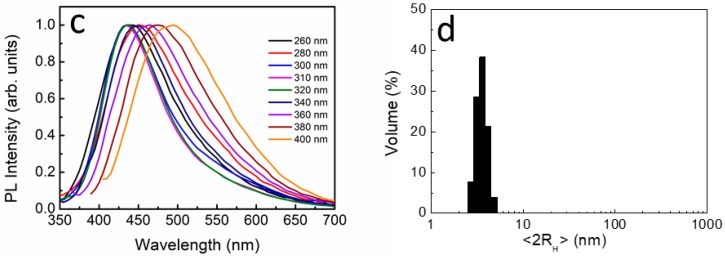
(**a**) Fluorescence and excitation (inset) spectra of the UFQDs aqueous suspension. Excitation and emission wavelengths were 300 nm and 445 nm, respectively. (**b**) UV-vis absorption spectrum of the UFQDs aqueous suspensions. (**c**) Emission spectra excited at different wavelengths. (**d**) Particle size distribution of UFQDs, determined by using dynamic light scattering: <2R_H_> 3.5 ± 1.0 nm.

Aqueous suspensions of UFQDs were prepared by oxidation and cage-opening of C_60_ buckminsterfullerene according to the reported method [45]. The obtained stable aqueous suspension appeared with the characteristic brownish colour, no appreciable Tyndall effect and UV-induced photoluminescence (Figure S1 of the Supporting Information). As displayed by TEM images (see Figure 1a,b) the UFQDs have a uniform dispersion without apparent aggregation and the size of the single nanoparticles ranges from 1 to 2.5 nm, in reasonable agreement with the hydrodynamic radius as determined by DLS (see below and Figure 2). The UFQDs also show similar FT-IR and ^13^C- NMR spectra (Figure 1c,d) as regular GOQDs [14,45,50,51] and the open-cage fullerene QDs of [45]. In particular, the FT-IR spectrum in Figure 1c clearly shows O–H stretching located around 3400 cm^−1^. The C=C vibration of sp^2^ carbon atoms was detected at 1600 cm^−1^. The peaks of graphene oxide at 1000 cm^−1^, 1150 cm^−1^, 1720 cm^−1^ and 1390 cm^−1^ are due to C–O, C–OH, C=O and C–O bonds, respectively. These structures prove the presence of carboxylic acid and hydroxyl groups. The NMR spectrum, on the other hand, exhibits the signals in the range 20–80 ppm due to aliphatic sp^3^ carbon atoms and those from 100 to 185 ppm corresponding to sp^2^ carbon atoms. The fluorescence and the excitation spectra (Figure 2a) were similar to those of [45] and, along with the absorption curve and the dependence of the emission peak on the excitation wavelength (Figure 2b,c), showed the characteristic spectral features found in regular GOQDs synthesized from graphite or citric acid [28,29,30,31,32,33,34,35]. In particular, the absorption spectrum (Figure 2b) exhibited the typical well-resolved peak at 230 nm, due to the π–π* transition of the aromatic sp^2^ domains, and a less pronounced shoulder at ~305 nm, which is generally attributed to the n-π* transition in C=O bonds of oxygen-containing functional groups [14]. This latter structure became a distinct peak at approximately 310 nm in the excitation spectrum (inset in Figure 2a) suggesting that a certain degree of light scattering affected the absorption data. Overall, these results indicate that the different lattice structure, i.e., the presence of carbon pentagons, does not alter the optical properties of UFQDs in comparison to regular GOQDs. DLS measurements (Figure 2d) confirmed that the as-prepared UFQDs had a relatively narrow size distribution with <2R_H_> 3.5 ± 1.0 nm, in agreement with the data of [45] and in reasonable agreement with the expected dimensions of monodisperse unfolded fullerene.

### 3.2. The Quenching Effect of Heavy Metal Ions on the PL of UFQDs

**Figure 3:**
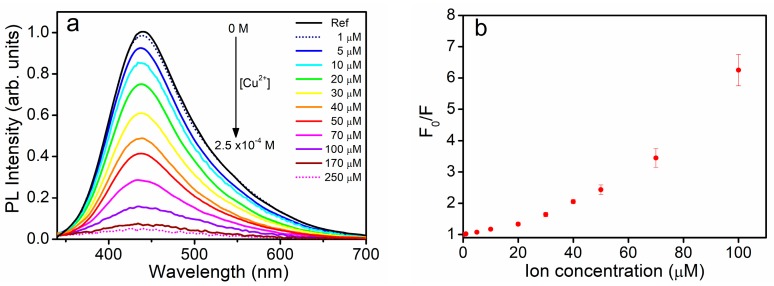
(**a**) Emission spectra of the reference aqueous suspension of UFQDs in the presence of Cu^2+^ at different concentrations. (**b**) Stern-Volmer plot describes the fluorescence quenching intensity vs. the Cu^2+^ concentration over the range 0–100 μM.

Several studies [28,29,30,31,32,33,34] have demonstrated that metal ions can interact with graphene quantum dots and produce quenching of the PL signal. In the present experiment, the aqueous solution of UFQDs exhibited a significant response to any of the following three heavy-metal ions: Cu^2+^, Pb^2+^ and As(III). As illustrated in Figure 3a, Cu^2+^ efficiently quenched the fluorescence signal F which gradually decreased with the increasing ion concentration without any appreciable change of the spectral profile. The quenching ratio F/F_0_ was linear versus the concentration of Cu^2+^ in the range 1–50 μM (see the calibration curve in Figure S2) and F approximately reached 16% of its initial value F_0_ at the concentration of 100 μM. The limit of detection (LOD) was calculated based on the IUPAC definition (3 σ of the reagent blank signal divided by the slope of the calibration curve [52]) and found to be 2 μM. The Stern-Volmer plot (Figure 3b) of the quenching experiment (F_0_/F versus [Cu^2+^]) showed the onset of a non-linear behaviour with a characteristic upward curvature also found in regular GOQDs [30], suggesting the occurrence of additional quenching mechanisms at high values of the ion concentration [53,54,55]. As will be discussed in the next paragraph, the decrease of the fluorescence was paralleled by a variation of the absorption spectrum of UFQDs. Very similar quenching response, absorption variations and Stern-Volmer plot were also obtained by Hao’s group by using regular GQDs [30].

**Figure 4:**
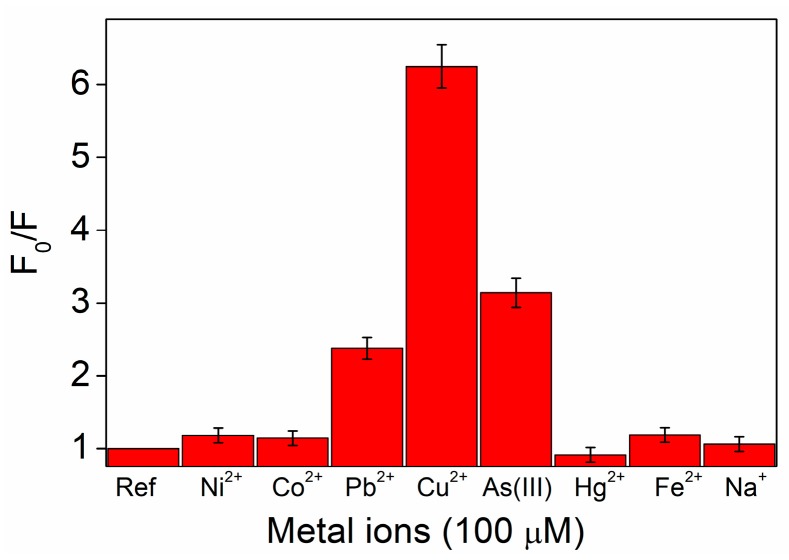
Fluorescence intensity ratio F_0_/F of the UFQD reference solution in the absence and presence of various individual metal ions at a concentration of 100 μM.

Comparable PL quenching effects, with different values of sensitivity (that is the slope of the calibration curve according to the IUPAC definition [52]), were also observed in the presence of Pb^2+^ and As(III) (Figures S3 and S4). The PL quenching with arsenic(III), however, showed a shorter range of linearity (down to 20 μM) which came along with a Stern-Volmer plot with an uncommon downward curvature (Figure S4b), thus suggesting a different quenching mechanism for this ion. This difference corresponded to a peculiar behaviour in the transmittance spectrum, as will be discussed in the next paragraph. Much lower or null response was measured with the other ions, as reported in Figure 4 which summarizes the fluorescence response F_0_/F of the UFQDs suspension to the different ions at the same concentration of 100 μM. 

It should be noted that a multiple response, i.e., sensitivity to more than one metal, was previously reported only in GOQDs prepared by chemical oxidation of carbon fibers [33,35] which demonstrated sensitivity to seven metal ions including Cu^2+^, similar to our study, but also Ni^2+^ and Co^2+^, which gave no response in the present experiment. Differently, in the rest of the literature [28,29,30,31,32,34] a selective quenching to a single ion has been reported. Within this framework, it should be pointed out that the sensitivity to Pb^2+^ observed in the present case is particularly interesting since it has never been reported in regular GQDs [29,30,32] except in the case of functionalization with specific organic groups [56,57]. This seems, therefore, a distinctive property of UFQDs in comparison with the GQDs studied so far. Finally, we note that, to our knowledge, this is first evidence of response to As(III) in a carbon-based quantum dot system. We note that the observed LOD of 2 μM for copper is far below the current World Health Organization limit of 30 μM in drinking water [38] and thus satisfactorily meets the first requirement for possible applications. On the other hand, the value of 2.5 μM measured for Pb^2+^ and As(III) is definitely much higher than the limits of 50 nM for lead and 14 nM for arsenic. However, similar exceeding values of LOD were reported in regular GOQDs [31,33]. Finally, in order to test the reproducibility of the multiple response to metal ions, with specific regard to the variability of the UFQD synthesis, the measurements of F_0_/F at 100 μM (see Figure 4) were repeated in four samples of UFQDs coming from different syntheses performed on distinct days. As reported in Figure S5 for the most relevant ions, the response varied within ±10% of the average value, approximately, which is quite satisfactory considering that the major contribution to fluctuations came from the intrinsic variability of the dialysis procedure carried out with a scientific laboratory set up. 

### 3.3. The Effect of Heavy-Metal ions on the Transmittance Spectrum and DLS Measurements

**Figure 5:**
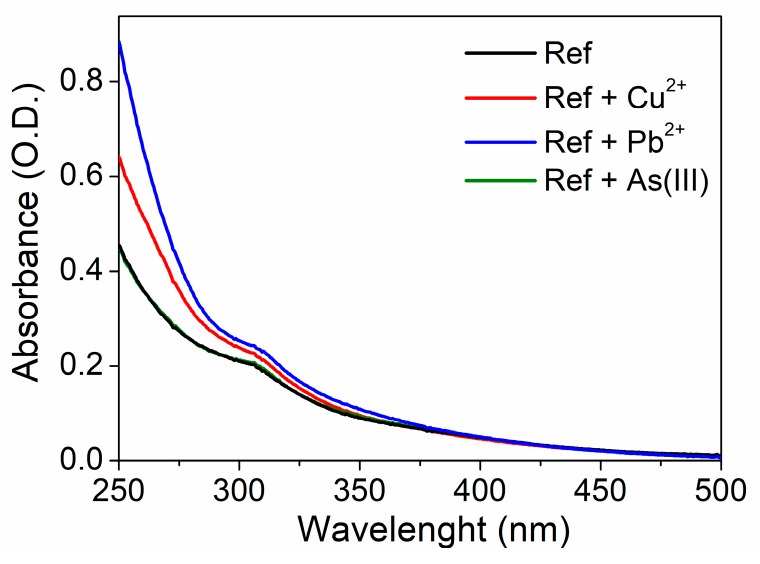
The UV-vis spectrum of the UFQD reference solution in the presence of Cu^2+^, Pb^2+^ and As(III) ions at a concentration of 100 μM.

In addition to PL quenching, the presence of Cu^2+^ and Pb^2+^ also produced modifications of the transmittance spectrum of the UFQD suspension, while no appreciable effects were reported with As(III) up to a concentration of 100 μM (Figure 5). Specifically, the presence of Cu^2+^ decreased the transmittance at shorter wavelengths, starting from 340 nm, with an appreciable smoothing of the peak at 300 nm due to the n-π* transition. In the case of Pb^2+^ the growth of apparent absorbance was significantly higher and started at longer wavelengths, with a slightly less smearing of the 300-nm peak. In both cases, the effect increased with the ion concentration similar to the PL quenching, i.e., approximately with a linear behaviour up to 50 μM (see Figures S6–S8). Neither appreciable blue shift of the peak, like in [30], nor clearly visible isosbestic points [31] were observed. The increment of apparent absorption cannot be ascribed to the absorbance of the metal ions in solution by themselves, since this effect is negligible from 250 to 500 nm (Figures S5–S7), which is the range which includes both the excitation and the emission wavelength of the present experiment. Conversely, the absorption of the metal ions is clearly visible below 250 nm and, as expected, correctly increases with ion concentration. In other words, the decrease of transmittance in the range 250–340 nm is necessarily produced by the interaction ion-UFQDs. In addition, we note that the UV-vis spectra were recorded with a 10-mm optical path, while the PL quenching measurements were carried out by using conventional 90° geometry and thus reducing to 1 mm the sample length experienced by both the excitation and the emission light. In this way, possible artefacts due to inner-filter effects and transmittance variations affecting the PL quenching experiment [33] can be ruled out. 

**Scheme 1:**
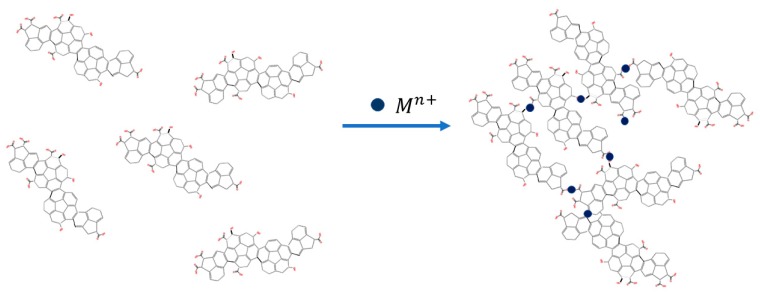
Hypothetical aggregation mechanism of UFQDs in the presence of metal ions.

The increment of apparent absorption with decreasing wavelength can rather be explained with Rayleigh light scattering due to formation of large UFQDs aggregates in the presence of metal ions. Aggregation could occur via multiple complexation, i.e., with the Cu^2+^ and Pb^2+^ ions binding more UFQDs together, by chelating with the –COOH and–OH groups located at the edges and on the surface of nanosheets [58], for instance with an edge-to-edge geometry as depicted in Scheme 1 [59].

**Figure 6:**
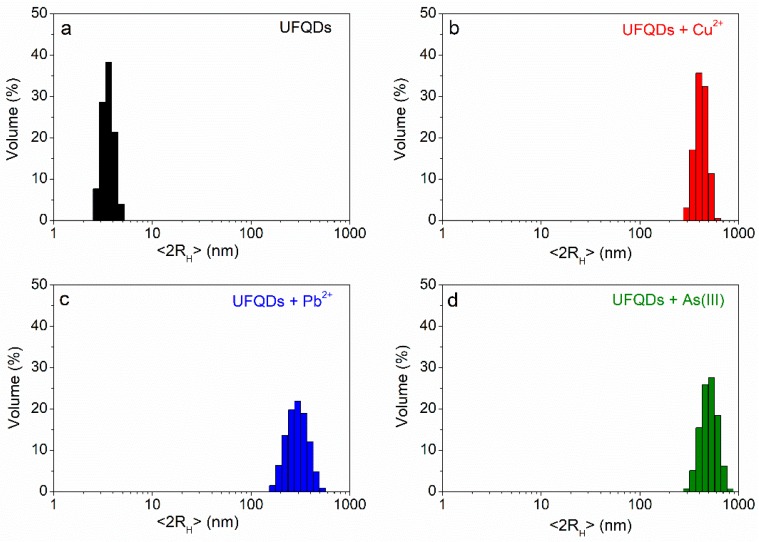
Particle size distribution of UFQDs as determined by dynamic light scattering: (**a**) in the absence of any ion: <2R_H_> = 3.5 ± 1.0 nm; (**b**) in the presence of 100 μM of Cu^2+^: <2R_H_> = 420 ± 25 nm; (**c**) in the presence of 100 μM of Pb^2+^: <2R_H_> = 300 ± 22 nm; (**d**) in the presence of 100 μM of As(III): <2R_H_> = 510 ± 35 nm.

Once an aggregate is formed, the fluorescent emission can be quenched both by energy transfer between the UFQDs and the chelated metal ion and by different radiationless mechanisms arising inside the aggregate itself. Nanosheet aggregation had already been suggested, without further analysis, as a possible explanation for the PL quenching in the presence of heavy-metal ions in GOQDs [32,33]. Very recently, multiple complexation has also been thoroughly characterized and discussed in GOQDs with mono- and divalent cations [60] (Na^+^, K^+^, Mg^2+^, and Ca^2+^) and taken into account to explain the increased interaction between graphene sheets [35]. In the present case, the formation of aggregates was confirmed by DLS measurements on the UFQD solution before and after the addition of the heavy-metal ions. The results (Figure 6) showed that the <2R_H_> of UFQD nanoparticles increased up to 300 ± 22 nm and to 420 ± 25 nm upon the addition of Pb^2+^ and Cu^2+^, respectively. 

Furthermore, TEM pictures taken after the addition of Cu^2+^ (see Figure S9) confirmed the absence of nanoparticles and the presence of aggregates with uneven shape and dimensions in the range of 100 nm, in reasonable agreement with the larger values of hydrodynamic radius measured by DLS. Finally, in the case of Pb^2+^, a brownish precipitate deposited at the bottom of the cuvette few hours after the addition to the UFQDs solution at concentration equal or higher than 100 μM (Figure S10). This indicated that aggregates had settled in the solution, possibly due to the higher atomic weight of lead, three times that of copper. Interestingly, no PL quenching was observed when the solution above the precipitated was added again to the UFQD reference solution (Figure S11), thus confirming that all the Pb^2+^ ions was bound to the aggregated UFQDs and dragged to the bottom of the container. In other words, UFQDs succeeded in efficiently removing lead(II) from the solution and this effect could also find application in purification of contaminated water.

**Figure 7:**
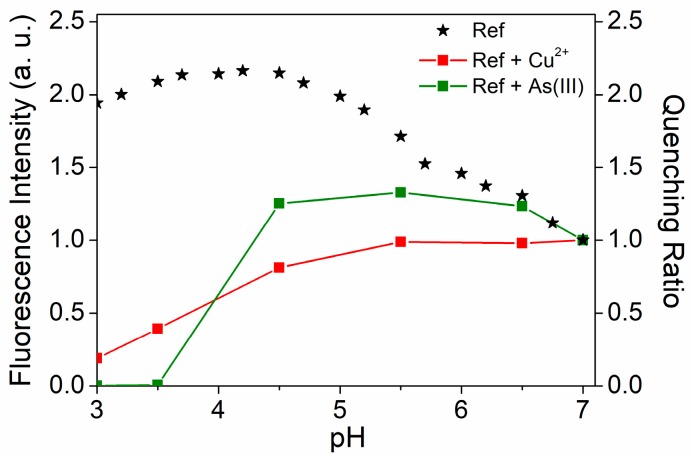
The fluorescence intensity F_0_ of the UFQD reference solution (black stars) and the fluorescence response F_0_/F in the presence of Cu^2+^ (red squares) and As(III) (green squares) at a concentration of 100 μM as a function of pH. All the data are normalized at the values at pH 7.

As mentioned above, the addition of arsenic did not alter the transmittance spectrum of UFQDs (Figure 5 and Figure S8), differently from Cu^2+^ and Pb^2+^. This corresponded to the peculiar Stern-Volmer plot of As(III) discussed in the previous paragraph (Figure S4b). On the other hand, DLS measurements showed the formation of aggregates in the presence of arsenic as well. We can speculate that As(III) induces a different type of aggregation, in comparison with Cu^2+^ and Pb^2+^, due to the different chemical reactivity and the presence in water of arsenic as oxy-compounds. In fact, as suggested by different studies [32,60], aggregation can occur either through functional groups located at the edges [59], such as the carboxyl groups (as depicted in Scheme 1), or by multiple interaction with the π states and –OH groups on the surface of nanosheets [58,59,61,62], thus giving rise to face-to-face aggregates with a different geometrical configuration (stacking). Further information about the quenching mechanisms could come from the dependence of the fluorescence processes on the pH of the solution where they occur. Figure 7 displays the fluorescence intensity of the reference UFQDs solution and the relative quenching ratio in the presence of Cu^2+^ and As(III) at a concentration of 100 μM as a function of pH. 

The measurements were performed at pH < 7, since metal ions form hydroxides and eventually precipitate in alkaline water conditions [63], and down to pH 3 to avoid self-aggregation of UFQDs due to the loss of surface charge [59,60,64]. The behaviour of fluorescence intensity of the reference UFQDs solution was similar to that found in regular GO, with a significant increase with decreasing pH down to pH 4 which was attributed to the progressive protonation of the carboxylic groups [64]. The decrease of intensity for more acidic conditions can be assigned to the arising of self-aggregation of UFQDs [59,60,64].

With regard to the response of UFQDs in the presence of the two metal ions, a substantial invariance of the quenching ratio was found for both the species in the range of pH 4.5–7. Below pH 4.5 the sensitivities to the two ions decrease with decreasing pH with different slope. The slow decrease of the signal from Cu^2+^ was similar to the pH dependence observed in the sorption of divalent metal ions by carbon nanotubes [65] and attributed to the competition between H^+^ and metal ions in binding to the carboxylic groups. The decrease in the presence of As(III) could be due to the fact that H^+^ bind with and neutralize the negative charge of the arsenic oxy-compounds, which thus would lose the capability to form complexes with UFQDs. In this case, since the negative charges of oxy-compounds are significantly fewer than the carboxylic groups of UFQDs, a faster decrease with increasing concentration of H^+^ is expected.

**Figure 8:**
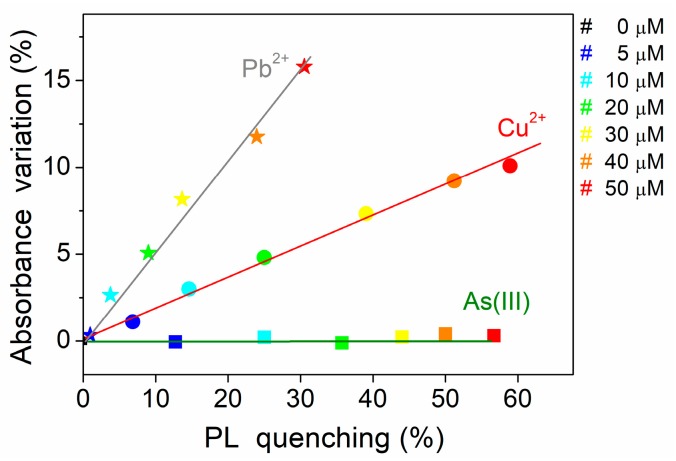
The relative increase of the apparent absorption, measured at λ_a_ = 275 nm, plotted as a function of the relative quenching ratio (F_0_ − F)/F_0_ for each ion at different the concentrations according to the color scale on the right. The lines are guides for the eye.

While further studies are needed to confirm this hypothesis the model of different types of aggregation, it should be pointed out that the different behaviour of the three ions, as regards the PL quenching and the transmittance variations, makes it possible to discriminate between the three species by using simple optical measurements. In fact, the ratio between the two effects varies across the three species, with the special case of As(III) which did not produce any variation of absorbance. On a more quantitative basis, this diversification can be represented in the multiple calibration diagram illustrated in Figure 8, where the relative increase of the apparent absorption, measured at λ_a_ = 275 nm, is plotted as a function of the quenching ratio (F_0_ − F)/F_0_ for each ion at different concentrations (the different colours). For the sake of simplicity, the figure only shows the data in the range of lower concentration values, i.e., up to 50 μM. 

The continuous lines reported in Figure 8 are guides for the eye which show that the different calibration curves do not cross each other because of the different behaviour of the three species. Therefore, the diagram demonstrates that the signature of a certain metal species at a specific concentration consists of a particular couple of values of the PL quenching and the absorbance variation. Conversely and consequently, for a single species contamination, a specific couple of values of the measured PL quenching and absorbance variation uniquely corresponds to a determined concentration of a certain metal species. We think this capability could be very promising in view of multiple selective detecting of heavy metals in aqueous media.

## 4. Conclusions

In summary, we have studied the optical response of UFQDs to some commonly encountered heavy-metal ions by recording the PL quenching, the variation of transmittance spectra and the hydrodynamic radius with DLS. The slightly different lattice structure of these novel carbon-based nanoparticles does not significantly alter the basic optical properties in comparison with those of typical regular GOQDs. On the other hand, the sensitivity to metal ions showed some peculiar characteristics. In particular, UFQDs exhibited a multiple PL-quenching response, with different characteristics, to any of the three ions: Pb^2+^, Cu^2+^ or As(III). The measured LOD satisfactorily meets the current limit for copper in drinking water and this makes UFQDs quite promising for applications to real sensing devices. On the other hand, LOD is about two order of magnitude higher the limits for lead and arsenic. While the use of a less noisy optical source, such as a light emitting diode (LED), could reduce the LOD appreciably, the increase of sensitivity by functionalization of UFQDs is mandatory in view of potential use in practical applications. By correlating fluorescence quenching with transmittance spectra, TEM images and DLS measurements, we could confirm, for the first time in carbon-based metal sensing, that aggregation of UFQDs is the key factor in the PL quenching response to all the three ions. However, aggregation seems to produce no modifications of transmittance in the case of As(III), while it leads to formation of precipitate in the presence of Pb^2+^. These differences clearly call for a deeper explanation and further studies are in progress to relate these effects to chelation with the different functional groups, electronic configuration and coordination characteristics of each of the involved metal ions. We note, however, that the distinct optical behaviour could make it possible to easily discriminate between the three metals for the implementation of a selective optical sensor. 

## Supplementary Materials

The following are available online at http://www.mdpi.com/1424-8220/17/11/2614/s1. Figure S1. Photograph of DI water (1) and UFQDs solution (2), taken under visible light (a) and 365-nm UV light (b). Figure S2. Fluorescence intensity ratio F/F_0_ of UFQDs as a function of Cu^2+^ concentration in the range 0–250 μM. Figure S3. (a) Emission spectra of UFQDs in the absence (Ref) and presence of Pb^2+^ at different concentrations; (b) Stern-Volmer plot describes the dependency of the quenching effect on the Pb^2+^ concentration in the range 0–100 μM. Figure S4. (a) Emission spectra of UFQDs in the absence (Ref) and presence of As(III) at different concentrations; (b) Stern-Volmer plot describes the dependency of the quenching effect on the As(III) concentration in the range 0–100 μM. Figure S5. The reproducibility of the quenching ratio in the presence of some relevant metal ions measured with UFQDs reference solutions prepared on different days. Figure S6. The UV-vis spectra of the UFQD reference solution in the presence of Cu^2+^ at different concentrations. It is also shown, for comparison, the absorption spectrum of the aqueous solution of the bare ion at 50 μM without UFQDs (dotted grey line). Figure S7. The UV-vis spectra of the UFQD reference solution in the presence of Pb^2+^ at different concentrations. It is also shown, for comparison, the absorption spectrum of the aqueous solution of the bare ion at 50 μM without UFQDs (dotted grey line). Figure S8. The UV-vis spectra of the UFQD reference solution in the presence of As(III) at different concentrations. It is also shown, for comparison, the absorption spectrum of the aqueous solution of the bare ion at 50 μM without UFQDs (dotted grey line). Figure S9. (a) Low-magnification, (b) medium-magnification and (c) high-magnification TEM images of UFQDs after the addition of Cu^2+^ ions a concentration of 100 μM. Figure S10. Photograph of the UFQDs solution taken 6 h after the addition of 100 μM Cu^2+^ (1), As(III) (2) and Pb^2+^ (3). Figure S11. Emission spectra of the UFQDs reference solution in the absence (black line), in the presence (red line) of 100 μM Pb^2+^ and in the presence of the Pb^2+^- quenched solution (blue line).
