# Supplementary material for: Sensitivity to Heavy-Metal Ions of Unfolded Fullerene Quantum Dots
Source: Sensors (Basel). 2017 Nov 14;17(11):2614. doi: 10.3390/s17112614 (PMC5713155; doi:10.3390/s17112614)
Supplement: Supplementary file 1 [file sensors-17-02614-s001.pdf]

Supplementary
Sensitivity to heavy-metal ions of unfolded fullerene quantum dots

Erica Ciotta [1], Stefano Paoloni [1], Maria Richetta [1], Paolo Prosposito [2], Pietro Tagliatesta [3], Chiara Lorecchio [3], Iole Venditti [4], Ilaria Fratoddi [4], Stefano Casciardi [5] and Roberto Pizzoferrato [1,*]

[1] Department of Industrial Engineering, University of Rome Tor Vergata, 00133 Rome, Italy; Erica.Ciotta@uniroma2.it (E.C.); stefano.paoloni@uniroma2.it (S.P.); richetta@uniroma2.it (M.R.)
[2] Department of Industrial Engineering INSTM and CiMER, University of Rome Tor Vergata, 00133 Rome, Italy; paolo.prosposito@uniroma2.it
[3] Department of Chemical Sciences and Technology, University of Rome Tor Vergata, 00133 Rome, Italy; pietro.tagliatesta@uniroma2.it (P.T.); ChiaraLorec@hotmail.it (C.L.)
[4] Department of Chemistry, University of Rome Sapienza, 00187 Rome, Italy; iole.venditti@uniroma1.it (I.V.); ilaria.fratoddi@uniroma1.it (I.F.)
[5] Department of Occupational & Environmental Medicine, National Institute for Insurance against Accidents at Work (INAIL), 00133 Rome, Italy; s.casciardi@inail.it
* Correspondence: pizzoferrato@uniroma2.it; Tel.: +39-0672-597-192; Fax: +39-0672-021-351

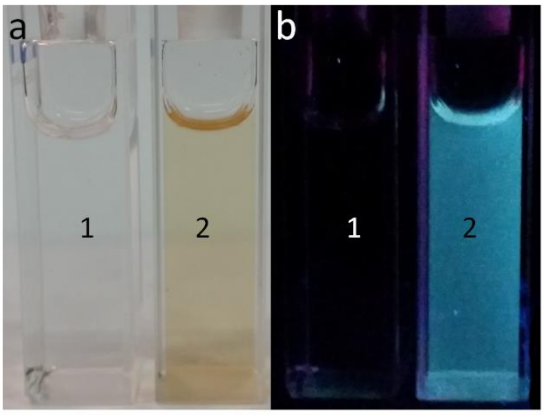

Figure S1. Photograph of DI water (1) and UFQDs solution (2), taken under visible light (**a**) and 365-nm UV light (**b**).

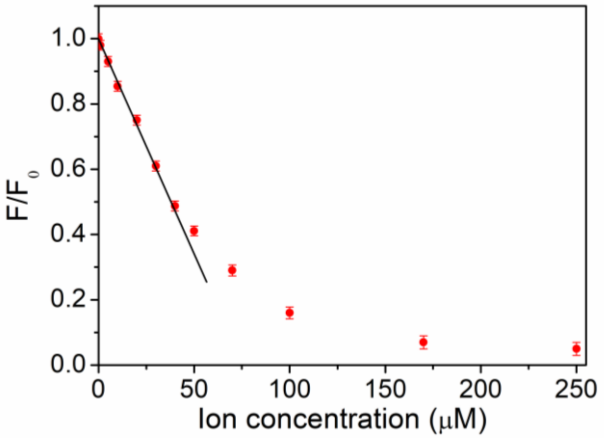

Figure S2. Fluorescence intensity ratio F/F_0 of UFQDs as a function of Cu^{2+} concentration in the range 0 – 250 μM

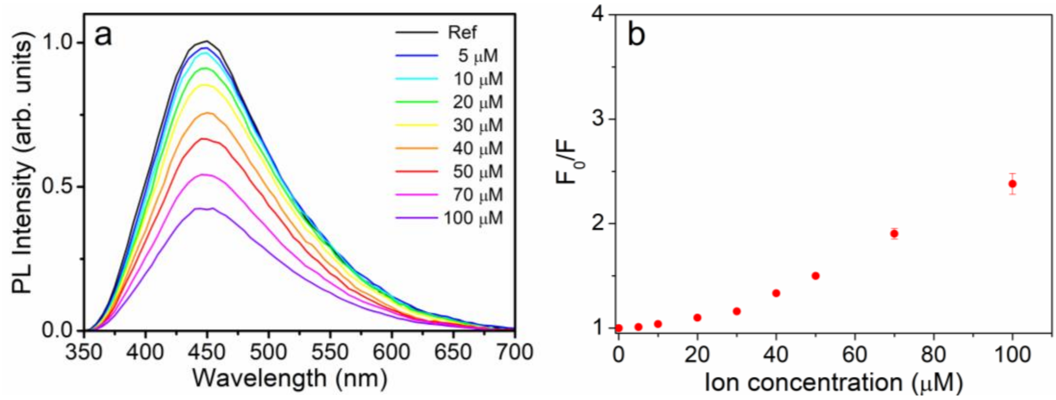

Figure S3. (**a**) Emission spectra of UFQDs in the absence (Ref) and presence of Pb^{2+} at different concentrations; (**b**) Stern-Volmer plot describes the dependency of the quenching effect on the Pb^{2+} concentration in the range 0-100 μM.

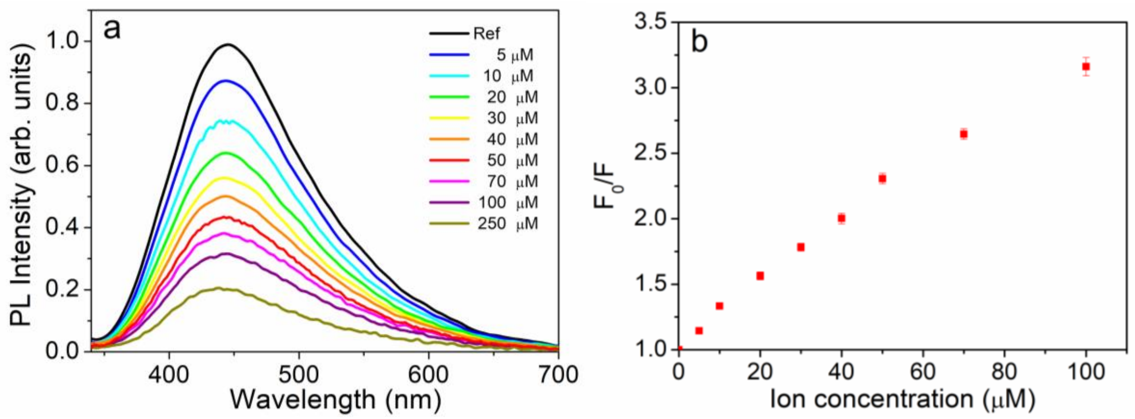

Figure S4. (**a**) Emission spectra of UFQDs in the absence (Ref) and presence of As(III) at different concentrations; (**b**) Stern-Volmer plot describes the dependency of the quenching effect on the As(III) concentration in the range 0-100 μM.

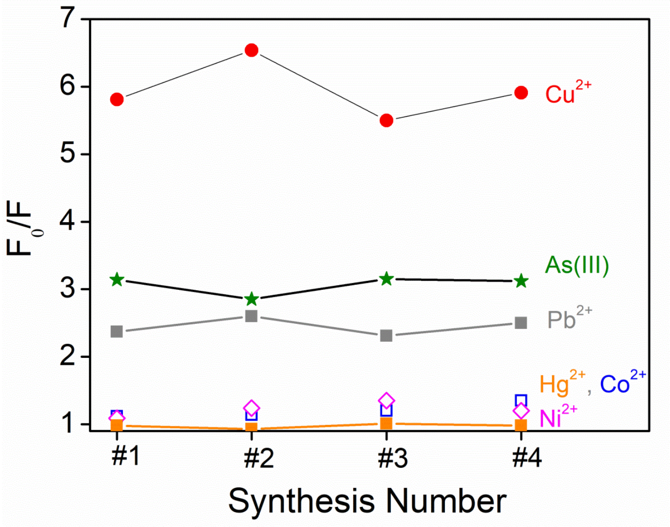

Figure S5. The reproducibility of the quenching ratio in the presence of some relevant metal ions measured with UFQDs reference solutions prepared on different days.

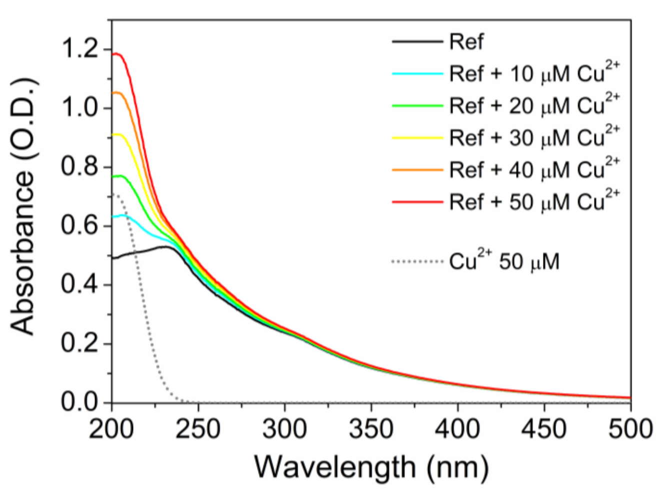

Figure S6. The UV-vis spectra of the UFQD reference solution in the presence of Cu^{2+} at different concentrations. It is also shown, for comparison, the absorption spectrum of the aqueous solution of the bare ion at 50 μM without UFQDs (dotted grey line).

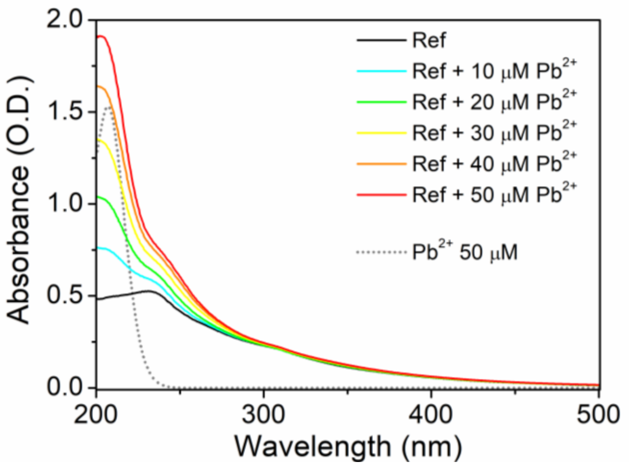

Figure S7. The UV-vis spectra of the UFQD reference solution in the presence of Pb^{2+} at different concentrations. It is also shown, for comparison, the absorption spectrum of the aqueous solution of the bare ion at 50 μM without UFQDs (dotted grey line).

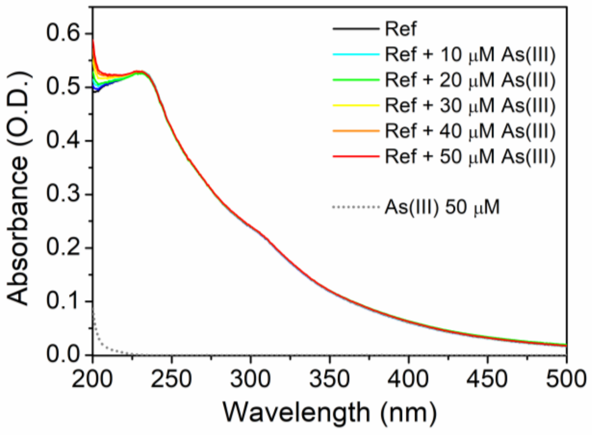

Figure S8. The UV-vis spectra of the UFQD reference solution in the presence of As(III) at different concentrations. It is also shown, for comparison, the absorption spectrum of the aqueous solution of the bare ion at 50 μM without UFQDs (dotted grey line).

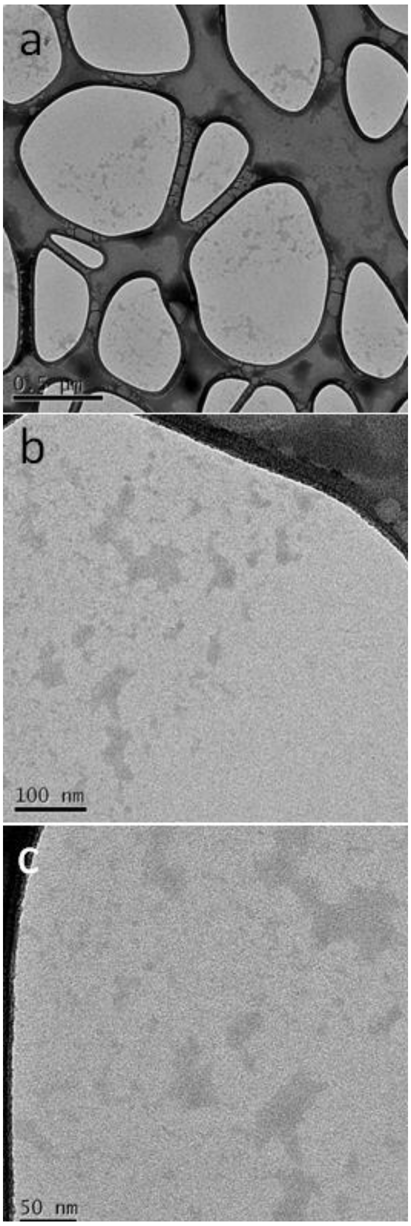

Figure S9. (**a**) Low-magnification, (**b**) medium-magnification and (**c**) high-magnification TEM images of UFQDs after the addition of Cu^{2+} ions a concentration of 100 μM.

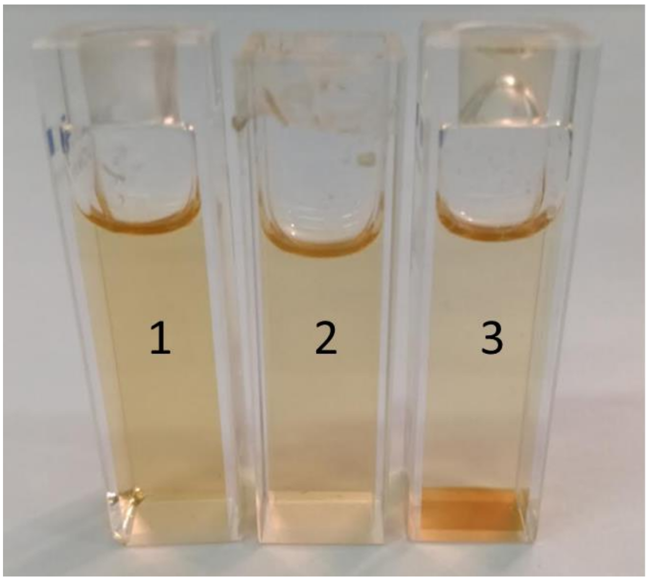

Figure S10. Photograph of the UFQDs solution taken 6 h after the addition of 100 μM Cu^{2+} (1), As(III) (2) and Pb^{2+} (3).

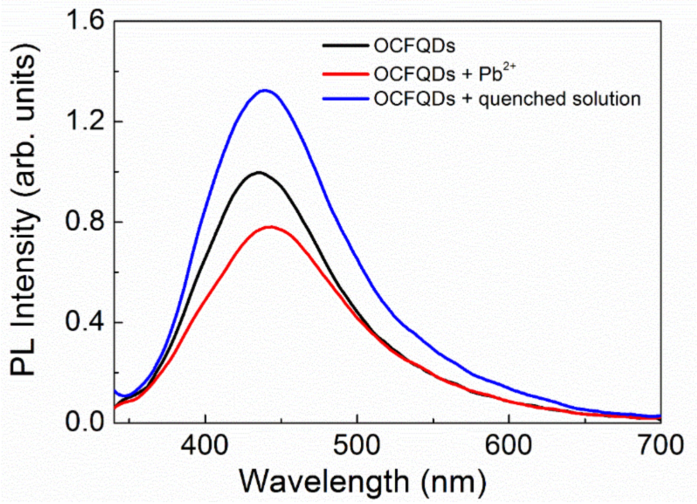

Figure S11. Emission spectra of the UFQDs reference solution in the absence (black line), in the presence (red line) of 100 μM Pb^{2+} and in the presence of the Pb^{2+}- quenched solution (blue line).

When the quenched solution was added, the emission intensity of the OCFQDs reference solution (blue line) increased above the reference value (black line), instead of being quenched, since new UFQDs were added in place of the Pb^{2+} ions.
